# Topology of the lateral visual system: The fundus of the superior temporal sulcus and parietal area H connect nonvisual cerebrum to the lateral occipital lobe

**DOI:** 10.1002/brb3.2945

**Published:** 2023-03-13

**Authors:** Nicholas B. Dadario, Onur Tanglay, Jordan F. Stafford, Ethan J. Davis, Isabella M. Young, R. Dineth Fonseka, Robert G. Briggs, Jacky T. Yeung, Charles Teo, Michael E. Sughrue

**Affiliations:** ^1^ Robert Wood Johnson Medical School Rutgers, The State University of New Jersey New Brunswick New Jersey USA; ^2^ Omniscient Neurotechnology Sydney New South Wales Australia; ^3^ Department of Neurosurgery University of Oklahoma Health Sciences Center Oklahoma City Oklahoma USA; ^4^ Cingulum Health Sydney New South Wales Australia; ^5^ Centre for Minimally Invasive Neurosurgery Prince of Wales Private Hospital Sydney New South Wales Australia

**Keywords:** dyslexia, FST, parcellation, PH, tractography, visual network

## Abstract

**Background and Purpose:**

Mapping the topology of the visual system is critical for understanding how complex cognitive processes like reading can occur. We aim to describe the connectivity of the visual system to understand how the cerebrum accesses visual information in the lateral occipital lobe.

**Methods:**

Using meta‐analytic software focused on task‐based functional MRI studies, an activation likelihood estimation (ALE) of the visual network was created. Regions of interest corresponding to the cortical parcellation scheme previously published under the Human Connectome Project were co‐registered onto the ALE to identify the hub‐like regions of the visual network. Diffusion Spectrum Imaging‐based fiber tractography was performed to determine the structural connectivity of these regions with extraoccipital cortices.

**Results:**

The fundus of the superior temporal sulcus (FST) and parietal area H (PH) were identified as hub‐like regions for the visual network. FST and PH demonstrated several areas of coactivation beyond the occipital lobe and visual network. Furthermore, these parcellations were highly interconnected with other cortical regions throughout extraoccipital cortices related to their nonvisual functional roles. A cortical model demonstrating connections to these hub‐like areas was created.

**Conclusions:**

FST and PH are two hub‐like areas that demonstrate extensive functional coactivation and structural connections to nonvisual cerebrum. Their structural interconnectedness with language cortices along with the abnormal activation of areas commonly located in the temporo‐occipital region in dyslexic individuals suggests possible important roles of FST and PH in the integration of information related to language and reading. Future studies should refine our model by examining the functional roles of these hub areas and their clinical significance.

## Graphical abstract

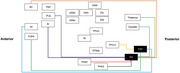

## 1 INTRODUCTION

Understanding the functional and structural connectivity of visual processing in the brain can provide further insight into how other complex cognitive processes occur. For instance, the processes of speech and reading require integration of visual and nonvisual information, which is thought to occur within the lateral occipital lobe (LOL), and relay visual information to nonvisual parts of the cerebrum (Bailey et al., 2018; Emberson et al., 2017). Describing the connectivity of the LOL is critical to understanding how the rest of the cerebrum accesses visual information, which in turn would advance our understanding of the visual system and disorders related to visual processing.

The anatomy and topological structure of the LOL is unclear and as such so is our understanding of visual processing. However, brain graphs and connectomic analyses of large sets of neuroimaging data have suggested a highly efficient architectural organization of brain networks, often containing nodes (or “hubs”), in order to efficiently facilitate complex functions while minimizing metabolic costs (Bullmore & Sporns, 2012). Equally, disruption of these brain hubs can prevent the effective integration of information across multiple functional systems necessary for complex functions (Dadario & Sughrue, 2022; Dadario et al., 2021). For instance, atypical functioning of hub regions necessary for the acquisition of reading and spelling skills is common in dyslexic patients (Bailey et al., 2018; Finn et al., 2014). Interestingly, some areas within the LOL have been similarly identified across dyslexia meta‐analyses, suggesting a possible additional role of the LOL in integrating information from language‐related networks (Bailey et al., 2018). Simultaneously, the visual word form area is also in this region, which computes representations of visually presented words, independent of their shape, size, font, or location (Dehaene & Cohen, 2011; Finn et al., 2014). This suggests the LOL is an important region for the visual system, but may also provide insight into the mechanisms behind multinetwork disturbances that underlie many language and reading disorders (Richards & Berninger, 2008).

In this study, we mapped the topology of the visual system based on the cortical parcellation scheme published under the Human Connectome Project (HCP) to understand how nonvisual areas access information in the LOL (Glasser et al., 2016). Through the use of coordinate‐based meta‐analytic connectivity modeling techniques on task‐based functional MRI (fMRI) literature, we analyzed patterns of coactivation across numerous healthy subjects to identify regions of interest (ROIs) that may serve as key hubs in the LOL to facilitate extravisual roles such as language and reading. Then, we sought to determine the structural interconnectedness of these ROIs with extravisual cerebrum through the use of deterministic tractography on healthy adult subjects to discuss how their structure may influence their known functional relevance. Our aim is to provide insight into higher visual networks with the ultimate goal of providing actionable anatomic information that can be utilized to guide clinical decision‐making in these regions, such as during cerebral surgery in the LOL to reduce neurologic deficits and also for novel target selection in neuromodulatory treatments.

## 2 METHODS

### 2.1 Creation of three‐dimensional ROIs

The three‐dimensional ROIs used in this study were generated from data previously published by the HCP (Glasser et al., 2016). In their study, the authors used surface‐based grayordinates to study 180 cortical ROIs. Grayordinate data were converted to volumetric dimensions in the Montreal Neuroimaging Institute (MNI) coordinate space using the Connectome Workbench command line interface (Van Essen Laboratory, Washington University 2016). A single ROI was generated for each of the parcellations as outlined by the HCP parcellation scheme (Glasser et al., 2016).

### 2.2 Literature search strategy and parcellations

We searched BrainMap Sleuth 2.4 (brainmap.org) on July 25, 2019 for all relevant task‐based fMRI studies related to the lateral occipital parcellations identified by the HCP authors (Glasser et al., 2016), including areas middle temporal (MT), medial superior temporal (MST), fundus of the superior temporal sulcus (FST), lateral occipital 1 (LO1), lateral occipital 2 (LO2), lateral occipital 3 (LO3), V3CD, V4t, and parietal area H (PH). The following search algorithm was used: (A) Locations: MNI Image, which prompts to upload the MNI ROI file created in Multi‐image Analysis GUI 4.0.1 (Mango; ric.uthscsa.edu/mango); (B) Subjects: Diagnosis = Normal. In total, we performed nine searches (one for each lateral occipital ROI) and returned 394 papers related to the MNI images of the lateral occipital parcellations constructed in Connectome Workbench. All papers were reviewed for inclusion in this study based on the following inclusion criteria: (1) peer‐reviewed publication, (2) task‐based fMRI study related to the visual network and vision (“Behavioral Domain”), (3) based on whole‐brain, voxel‐wise imaging, (4) including standardized coordinate‐based results in the Talairach or MNI coordinate space, and (5) including at least one healthy human control cohort. Only coordinates from healthy subjects were utilized in our analysis.

By first including studies related to the Behavioral Domain of vision, our search was able to capture a large amount of studies where activated foci were localized in the LOL, regardless of different study designs employed or the broad terminology utilized for indexing a study in services like PubMed. Then, in order to focus our searches on specific connections the lateral occipital parcellations demonstrated outside the visual network, papers discovered to cite the ROI were eliminated if they were related to the following Behavioral Paradigms: Perception/Vision, Visual Object Identification, Face Monitoring/Discrimination, Visual Pursuit/Tracking, Visual Motion, or Visuospatial attention (brainmap.org/taxonomy/paradigms.html) (Laird et al., 2009).

BrainMap contains a coding scheme with a rigorous taxonomy that incorporates structured keywords that elucidate the nature of each experimental design in the included studies. First, with the inclusion of broad Behavioral Domains, we were able to identify important cortical regions in the visual network. Following this initial search, incorporation of more specific Behavioral Paradigms filters that excluded studies solely related to visual processing then allowed us to further focus our search on nonvisual, task‐dependent coactivation patterns demonstrated by regions in the visual network according to the literature. Ultimately, while we removed studies focused solely on visual processing, these studies were still screened in detail and if they focused on extravisual functioning, then they were considered for inclusion in our subsequent analyses. Therefore, this comprehensive search strategy facilitates a rapid, yet comprehensive retrieval of relevant results, with noted limitations (Eickhoff et al., 2009; Laird et al., 2009). Overall, 142 papers met criteria for inclusion in this study (Table 1).

**TABLE 1:** Table of 142 included studies. Information on the study name, number of participants, and Montreal Neuroimaging Institute coordinates is included

Reference	Number of participants	Number of foci
Area FST		
Etard et al., 2000	9	13
Fiez et al., 1999	11	31
Drobyshevsky et al., 2006	31	14
Mummery et al., 1996	6	2
Thompson et al., 2007	17	25
Bocher et al., 2001	10	6
Lowell et al., 2008	14	26
Wright et al., 2004	8	3
Mickley & Kensinger, 2008	20	5
Shah et al., 2013	28	73
Anderson et al., 2004	24	38
Aoki et al., 2005	10	12
Area LO1		
Buckner et al., 1998	26	53
Kelley et al., 1998	5	19
Milham et al., 2003	11	28
Landau et al., 2004	10	5
Kuo et al., 2004	10	16
Saccuman et al., 2006	13	6
Fehr et al., 2008	11	17
Mohamed et al., 2006	6	9
Kensinger & Schacter, 2006b	21	8
Suzuki et al., 2001	6	2
Tricomi et al., 2009	15	13
Habeck et al., 2004	18	25
Bottini et al., 2001	3	45
Nahab et al., 2009	18	15
Area LO2		
Banich et al., 2000	16	9
Suzuki et al., 2002	21	19
Uchida et al., 1999	2	10
Mochizuki‐Kawai et al., 2006	15	9
Poldrack et al., 2005	13	7
Tham et al., 2005	6	14
Haller et al., 2007	16	14
Bocher et al., 2001	10	10
Sabatinelli et al., 2007	22	10
Davis et al., 2009	37	48
Ye & Zhou, 2009	19	14
Ghahremani et al., 2010	16	8
van Duinen et al., 2008	10	25
Knutson & Greer, 2008	24	5
Talmi et al., 2008	11	17
Bottini et al., 2001	6	56
Kucian et al., 2006	20	10
Jeon et al., 2009	16	13
Parsons et al., 2005	12	16
Area LO3		
Ueda et al., 2003	15	8
Jenkins et al., 1994	12	22
de Zubicaray et al., 2000	8	37
Temple et al., 2001	15	20
Takahashi et al., 2006	11	6
Dick et al., 2007	12	19
Filimon et al., 2007	15	35
Rilling et al., 2007	22	5
Kensinger & Schacter, 2006a	21	18
Garrett & Maddock, 2006	9	29
Guillot et al., 2009	50	9
Lissek et al., 2008	13	15
Harenski et al., 2008	14	4
Habeck et al., 2004	18	25
Talmi et al., 2008	11	10
Gutchess & Schacter, 2012	16	4
Harenski et al., 2010	16	15
Carter et al., 2012	13	28
Wylie et al., 2006	13	19
Raboyeau et al., 2010	10	10
Pornpattananangkul et al., 2018	31	7
Gu et al., 2015	17	22
Cascio et al., 2012	14	6
Berlingeri et al., 2010	24	8
Soloveva et al., 2020	15	48
Area MST		
Stevens et al., 2000	10	20
Landau et al., 2004	10	7
Kensinger & Schacter, 2007	19	1
Hennenlotter et al., 2005	12	20
Pierno et al., 2008	12	12
Addicott et al., 2012	13	19
Lotze et al., 2007	14	17
Area MT		
Gandour et al., 2003	10	6
Saccuman et al., 2006	13	5
Stark et al., 2007	66	11
Filimon et al., 2007	15	35
Wrase et al., 2003	10	8
Lee et al., 2006	18	28
Harenski et al., 2008	28	4
Langleben et al., 2009	18	4
Area PH		
Iidaka et al., 2000	12	22
Calvert & Campbell, 2003	8	52
Decety et al., 1994	6	64
Hasson et al., 2002	13	3
Rypma et al., 1999	6	41
Mazard et al., 2002	6	52
Tan et al., 2000	6	20
Rossell et al., 2002	12	11
Rossell et al., 2001	12	11
Chee et al., 2001	9	6
Peng et al., 2003	8	8
Vingerhoets et al., 2003	12	13
Gitelman et al., 1999	12	16
LaBar et al., 1999	11	26
Votaw et al., 1999	15	16
Fassbender et al., 2004	21	21
Nakamura et al., 2002	9	30
Polk & Farah, 2002	1	2
Siok et al., 2004	8	25
DiGirolamo et al., 2001	8	85
Brass & von Cramon, 2004	14	4
Dreher & Berman, 2002	14	7
Linden et al., 2003	12	2
Rypma et al., 2001	6	41
Bedwell et al., 2005	14	24
Baciu et al., 2005	10	9
Stark et al., 2007	66	15
Hofer et al., 2007	21	13
Harrington et al., 2007	11	38
Kensinger & Schacter, 2007	19	71
Thompson et al., 2007	17	25
Pihlajamäki et al., 2003	12	15
Blair et al., 2006	21	4
Bocher et al., 2001	10	9
Johnson & Rugg, 2007	16	3
Ferretti et al., 2005	10	18
Garrett & Maddock, 2006	9	29
Ghosh et al., 2008	10	37
Lowell et al., 2008	14	26
Moulier et al., 2006	10	24
Altamura et al., 2007	18	40
Ye & Zhou, 2009	19	6
Peters & Büchel, 2009	22	54
Remijnse et al., 2005	27	22
Malik et al., 2008	20	19
Desai et al., 2006	25	31
Bell‐McGinty et al., 2004	19	8
Habeck et al., 2004	18	25
Chee et al., 2003	8	7
Wong et al., 2011	19	33
Paulesu et al., 2003	8	15
Bedny & Thompson‐Schill, 2006	13	11
Kong et al., 2005	16	21
Shah et al., 2013	28	63
Anderson et al., 2004	24	38
Abutalebi et al., 2007	12	19
Atzil et al., 2011	23	21
Brendel et al., 2011	20	60
Monaco et al., 2015	11	7
Robertson et al., 2015	16	73
Choi et al., 2017	15	25
Lacourse et al., 2005	54	16
Area V3CD		
Casey et al., 1998	8	21
Cohen et al., 2003	9	6
DiGirolamo et al., 2001	8	68
Assaf et al., 2006	18	17
Talmi et al., 2008	11	7
Area V4t		
Mechelli et al., 2000	6	49
Banich et al., 2000	16	9
Simpson et al., 2000	18	41
Podzebenko et al., 2005	16	15
Milham et al., 2003	16	25
Mochizuki‐Kawai et al., 2006	15	9
Meyer et al., 2005	12	6
Tham et al., 2005	6	14
Lissek et al., 2007	33	21
Killgore et al., 2003	13	5
Filimon et al., 2007	15	14
Junghöfer et al., 2006	18	14
Redcay et al., 2008	20	50
Bottini et al., 2001	6	50
Grèzes et al., 2007	16	17
Nahab et al., 2009	18	5
Kucian et al., 2006	20	16
Pekkola et al., 2006	10	8

Abbreviations: FST, fundus of the superior temporal sulcus; MNI, Montreal Neuroimaging Institute; MT, middle temporal; PH, parietal area H.

### 2.3 ALE generation and identification of relevant cortical regions

Coordinate‐based activation likelihood estimation (ALE) meta‐analytic processing is performed on groups of coordinates to identify coactivation patterns and task‐dependent connectivity for a user‐defined ROI (Robinson et al., 2010). ALE analyses conservatively estimate regions of convergence based on the probability of an event occurring at each brain voxel. Together, ALE models and precisely coded databases like BrainMap allow the procuring of large amounts of foci from the literature to analyze and identify patterns of coactivation across large amounts of subjects (Chu et al., 2015; Eickhoff et al., 2009; Laird et al., 2009). Such methodology has been reiteratively applied and reproduced by our team on other visual areas and allows for detailed anatomic characterization with high precision (Allan et al., 2019; Briggs et al., 2021; Milton et al., 2021).

In the current study, we used BrainMap Ginger ALE 2.3.6 (brainmap.org) to extract the relevant fMRI data from all 142 studies used in this analysis for creation of an ALE map. All Talairach coordinates identified during literature review were converted to the MNI coordinate space using SPM Conversion in Ginger ALE. We subsequently performed a Single Study analysis using Cluster‐Level Interference in the MNI coordinate space (cluster level of 0.05, threshold permutations of 1000, uncorrected *p*‐value of .001). The ALE coordinate data were displayed on an MNI‐normalized template brain using the Mango 4.0.1. A code was used to generate centroids according to the center coordinates and areas reported by Ginger ALE, identify HCP parcellations overlapping with this centroid, and calculate the percentage overlap between the centroid and HCP parcellation. Nonvisual parcellations with a percentage overlap greater than 10% were included in our analysis (Table 2). The preconstructed ROIs of the parcellations were also overlaid on the ALE and compared visually to ensure true overlap with the ALE.

**TABLE 2:** Overlap between Human Connectome Project parcels and the identified activation likelihood estimation. Parcels that had an overlap >10% and that were not occipital visual regions have been bolded. Parcels are defined according to the Human Connectome Project Glasser Atlas

Region	Percentage overlap with ALE
FST ALE	
L_FST	0.22234157
L_PH	0.37571998
L_FFC	0.00496278
L_V4t	0.04553734
L_PIT	0.10948905
L_LO2	0.19831224
**L_FOP4**	0.29501916
L_MI	0.00821596
L_FOP5	0.05328377
**L_44**	0.1129019
L_45	0.00146681
L_24dd	0.03410582
L_24dv	0.02868852
L_p24pr	0.04042348
**L_a24pr**	0.14168618
**L_p32pr**	0.12662338
L_24dv	0.0136612
**L_TPOJ1**	0.13175723
**L_STSdp**	0.11327434
**L_6r**	0.14689548
PH ALE	
**L_Thalamus**	0.40684619
**L_24dd**	0.10668999
L_p24pr	0.01564875
**L_23d**	0.44508671
**L_a24pr**	0.53395785
**L_PFcm**	0.20243902
**L_RI**	0.13997628
**L_24dv**	0.87568306
**L_p32pr**	0.495671
L_SCEF	0.00132406
**L_23c**	0.22535211
**L_Caudate**	0.49238579
L_Pallidum	0.07494647
L_Putamen	0.04650483
L_POS1	0.03053435
L_V1	0.03647462
L_ProS	0.84231537
L_DVT	0.03983402
L_V3	0.0119216
L_VMV1	0.43966713
L_V2	0.00532258
L_V4	0.07225888
L_Hippocampus	0.00586455
L_H	0.04606241
L_VMV2	1
L_PHA1	0.0025
L_VVC	0.13234614
L_VMV3	0.68463074
**L_PHA3**	0.21440536
**L_PHA2**	0.15254237
L_MT	0.17681159
L_MST	0.57142857
L_FST	0.41997852
L_PH	0.40097475
L_PGi	0.00749496
L_TPOJ1	0.08652082
L_TE2p	0.07185406
L_FFC	0.04869727
L_TPOJ2	0.09176471
L_V4t	0.00364299
L_PIT	0.00625652
L_LO2	0.06540084
**L_STSdp**	0.11327434
L_STSvp	0.01581509
L_PHT	0.00351741
**L_IFJp**	0.30054897
**L_8C**	0.26848756
**L_6r**	0.10254879
MT ALE	
L_MST	0.38095238
L_FST	0.33727175
L_MT	0.76811594
L_LO3	0.02356406
L_V4t	0.03460838
L_TPOJ2	0.0054902
L_V4	0.00596921
MST ALE	
L_MST	0.23511905
L_FST	0.31901182
L_PH	0.05804165
L_MT	0.02318841
L_V4t	0.03460838
L_LO2	0.03164557
V3CD ALE	
L_V3CD	0.21571649
L_LO1	0.14226519
L_PGp	0.03263274
L_LO3	0.06921944
L_MST	0.00297619
V4t ALE	
L_V4	0.00848256
L_V8	0.04845815
L_MST	0.17559524
L_FST	0.30504834
L_PIT	0.40250261
L_FFC	0.03349876
L_PH	0.10899424
L_V4t	0.43169399
L_MT	0.03478261
L_LO2	0.39135021
L_PH	0.01772264
L_FST	0.04081633
L_MST	0.0327381
**L_AIP**	0.17647059
LO1 ALE	
L_V4	0.1420044
L_V3	0.00101031
L_V8	0.25286344
L_VMV3	0.11377246
L_VVC	0.00185099
L_FFC	0.01830025
L_MST	0.16666667
L_FST	0.14822771
L_PIT	0.16058394
L_PH	0.07620735
L_V4t	0.25865209
L_LO2	0.07172996
LO2 ALE	
L_V4	0.1683946
L_V8	0.15859031
L_V3	0.00424328
L_LO1	0.02762431
L_FFC	0.06296526
L_FST	0.05585392
L_PIT	0.44421272
L_MST	0.04464286
L_V4t	0.33879781
L_PH	0.01550731
L_LO2	0.40822785
**L_LIPd**	0.29020979
L_LIPv	0.04328224
**L_AIP**	0.29838022
**L_IP2**	0.39013453
L_PFm	0.00573576
L_7 PC	0.02969469
L_24dd	0.01923918
L_SCEF	0.00926845
L_24dv	0.06693989
L_p32pr	0.0508658
LO3 ALE	
L_TPOJ3	0.99410609
**L_PGp**	0.14878319
L_LO3	0.99410898
L_MT	1
L_V3CD	0.00308166
L_LO1	0.1961326
L_MST	1
L_FST	0.98818475
**L_PGi**	0.27788988
L_V4t	0.72495446
L_PH	0.19937971
**L_TPOJ2**	0.94901961
L_TPOJ1	0.05010586
L_PGs	0.00572701
L_LO2	0.12763713
L_STV	0.05925098
**L_PHT**	0.23214914

### 2.4 Network tractography

Functionally connected regions of a network tend to be structurally connected. This idea is supported by both experimental and computational data and has been demonstrated by our team in relevant cortices well (Allan et al., 2019; Baum et al., 2020; Briggs et al., 2021; Bullmore & Sporns, 2012; Honey et al., 2007; Kaiser & Hilgetag, 2006; Sheets et al., 2021; White et al., 1986). Specifically, network analyses partly depend on the observations that the function of a neural node is in part determined by its structural interconnectedness with other nodes in the network and that structural analyses may place important constraints on the possibilities of functional relatedness between cortical regions (Bullmore & Sporns, 2012). This is further shown by the observation that structural and functional networks may share common modules and hubs (Bullmore & Sporns, 2012). Thus, while there are some limitations to the idea that structural and functional networks are interconnected, we proceeded to determine the backbone of the network using deterministic tractography.

All fiber tractography was completed in DSI Studio (http://dsi‐studio.labsolver.org) using publicly available brain imaging from the HCP (http://humanconnectome.org; release Q3). The acquisition parameters for the HCP data are available within the HCP documentation. Tractography was performed individually with 50 adult subjects, chosen randomly using a random number generator. The inclusion and exclusion criteria for the HCP database have been described elsewhere (Van Essen et al., 2013). Subjects had a mean age of 28.4 years and were mixed between nearly equal females (51%) and males (49%). A multishell diffusion scheme was used, with *b*‐values of 1000, 2000, and 3000 s/mm^2^. Each *b*‐value was sampled in 90 directions. The in‐plane resolution was 1.25 mm and the slice thickness was 1.25 mm. The diffusion data were reconstructed using generalized *q*‐sampling imaging with a diffusion sampling length ratio of 1.25. All reconstructions were performed in MNI space using an ROI approach to initiate fiber tracking from a seeded region (areas FST and PH). Grayordinate label parcellation fields were standardized to the three‐dimensional volumetric working spaces of DSI studio using the structural imaging data provided by HCP for each subject. Voxels within each ROI were automatically traced with a maximum angular threshold of 45°. When a voxel was approached with no tract direction or a direction greater than 45°, the tract was halted. Tracts with length shorter than 20 mm or longer than 800 mm were discarded. In some instances, exclusion ROIs were placed to exclude spurious tracts or tracts inconsistently represented across individuals.

### 2.5 Measuring connection strength

DSI studio was used to generate an adjacency matrix of connections between different ROIs to quantify strength between different cortical regions. The tracts were generated using 2.5 million randomly placed seeds. Working sequentially through ROI pairs in the network, the number of tracts between regions was recorded for each subject after fiber tractography was terminated under these new conditions. Tracts between parcellations were considered significant if they could be identified consistently in five or more subjects. The strengths of the connections within the network were calculated by averaging the number of tracts between each ROI pair of the network across all subjects.

## 3 RESULTS

### 3.1 FST and PH have the greatest coactivation with extraoccipital areas

**FIGURE 1:**
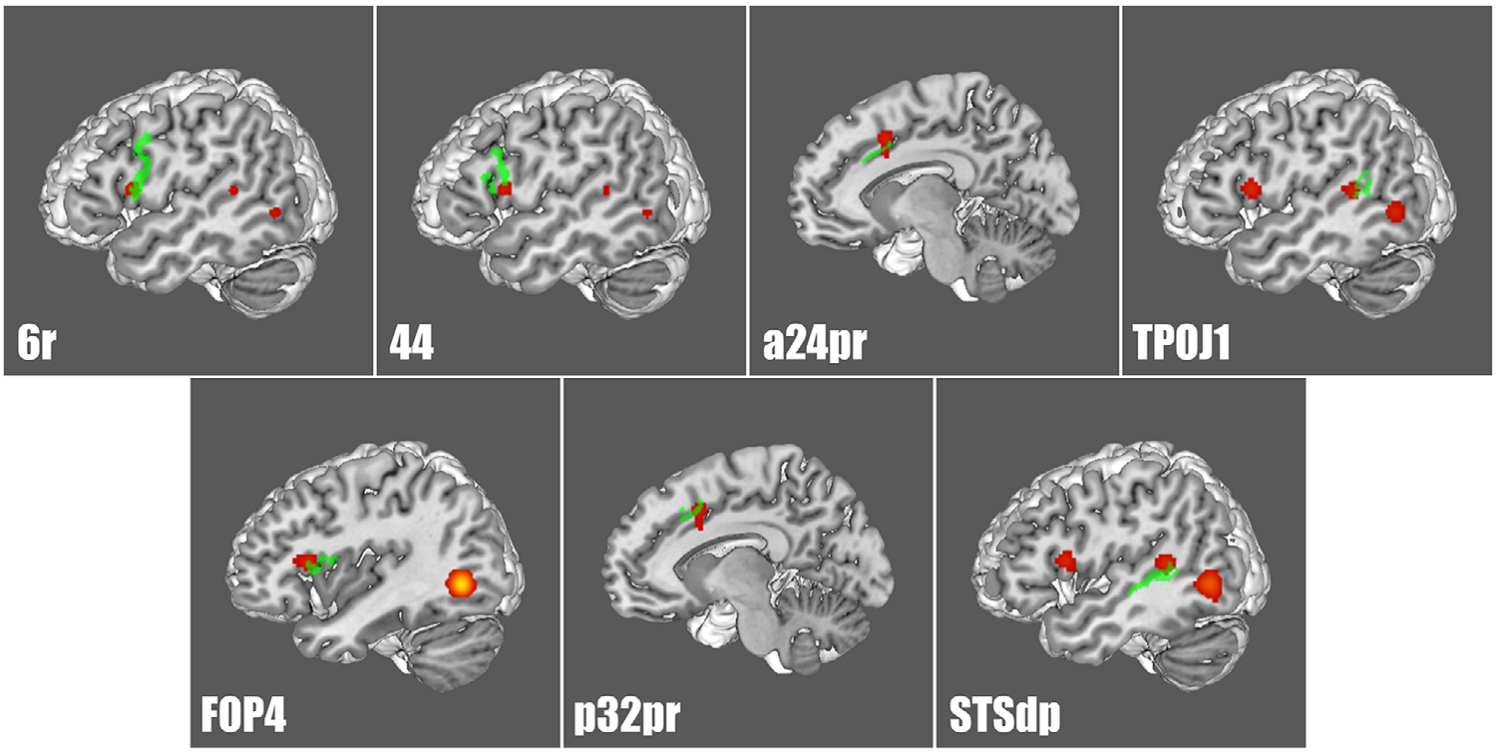
Activation likelihood estimation of task‐based functional MRI experiments related to area FST. The three‐dimensional ALE data (red) are displayed in Mango on a brain normalized to the Montreal Neuroimaging Institute coordinate space, with overlapping HCP parcellations (green). Here, ALE data represents areas that coactivate with FST. ALE, activation likelihood estimation; FST, fundus of the superior temporal sulcus; 6r, rostral premotor area 6; a24pr, anterior 24 prime; p32pr, posterior 32 prime; TPOJ1, temporo–parieto–occipital junction 1; FOP4, frontal opercular area 4.

**FIGURE 2:**
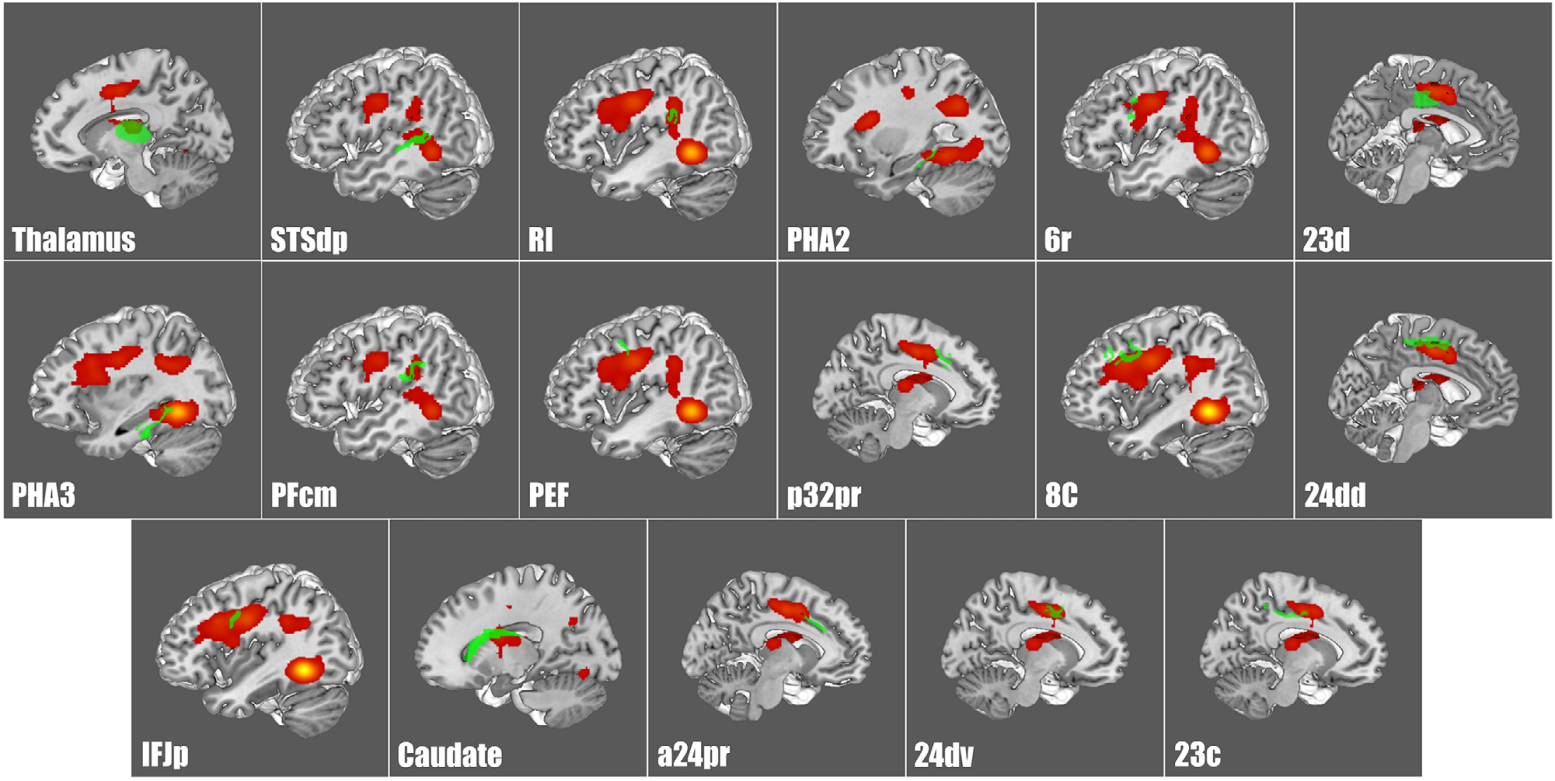
Activation likelihood estimation of task‐based functional MRI experiments related to area PH. The three‐dimensional ALE data (red) are displayed in Mango on a brain normalized to the Montreal Neuroimaging Institute coordinate space, with overlapping Human Connectome Project parcellations (green). Here, ALE data represent areas that coactivate with PH. ALE, activation likelihood estimation; 8c, Brodmann area 8 component C; PEF, prefrontal eye field; IFJp, inferior frontal junction area/posterior; 6r, rostral premotor area 6; p32pr, posterior 32 prime; a24pr, anterior 24 prime; 24dv, ventral area 24d; 24dd, dorsal area 24d; 23c, Brodmann area 23 component C; 23d, Brodmann area 23 component D; PFcm, Brodmann area 40 PFcm complex; RI, retroinsular; STSdp, superior temporal sulcus dorsal posterior; PHA2, parahippocampal area 2; PHA3, parahippocampal area 3.

**FIGURE 3:**
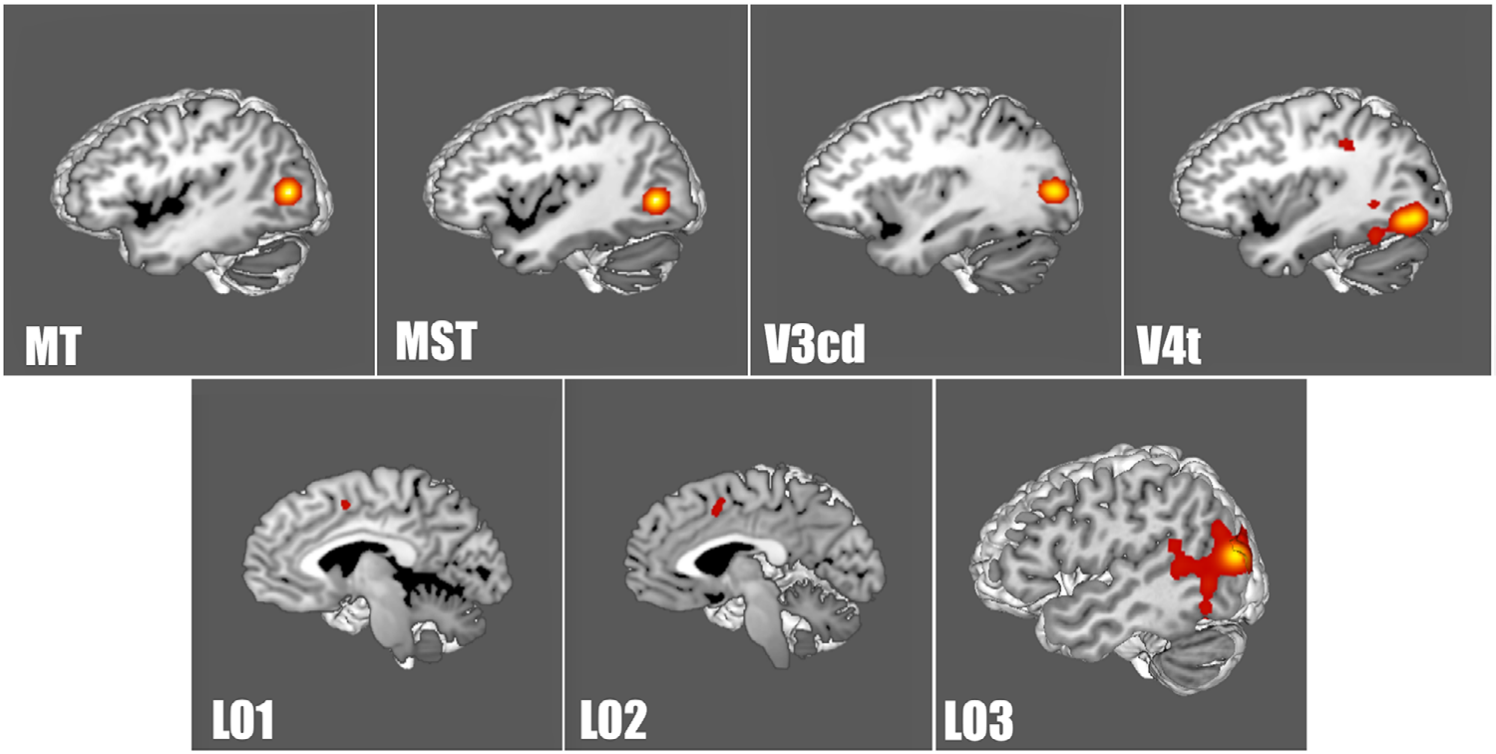
Activation likelihood estimation of task‐based functional MRI experiments related to lateral occipital lobe parcellations: areas middle temporal, medial superior temporal, fundus of the superior temporal sulcus, lateral occipital 1, lateral occipital 2, lateral occipital 3, V3CD, V4t, and parietal area H. The three‐dimensional ALE data (red) are displayed in Mango on a brain normalized to the Montreal Neurological Institute coordinate space. ALE, activation likelihood estimation; MT, middle temporal; MST, medial superior temporal; LO, lateral occipital.

A Sleuth search and GingerALE meta‐analysis on all of the lateral occipital parcellations defined by Glasser et al. allowed us to determine their relative coactivations during fMRI studies outside the visual network (Glasser et al., 2016). Table 2 lists the HCP parcellations that matched to each ALE. FST and PH exhibited the greatest coactivation during task‐based fMRI studies to parcellations outside of the visual network (Figures 1 and 2). Figures 1 and 2 demonstrate the three‐dimensional ALE data in relation to FST and PH. Differently, the remaining lateral occipital ROIs (MT, MST, V3cd, V4t, LO1, LO2, and LO3) demonstrated less extensive coactivation outside of the lateral visual network (Figure 3).

When just considering FST and PH ALEs, they each exhibited unique connections to nonvisual cortices. The ALE of area FST demonstrated coactivation with areas 44 (Brodmann area 44), 6r (rostral premotor area 6), FOP4 (frontal operculum 4), TPOJ1 (temporo–parieto–occipital junction 1), a24pr (anterior 24 prime), p32pr (posterior 32 prime), and STSdp (superior temporal sulcus dorsal posterior) (Figure 2). Additionally, the ALE of area PH exhibited coactivation with areas 23c (Brodmann area 23 component C), 23d (Brodmann area 23 component D), 24dv (ventral area 24d), 24dd (dorsal area 24d), a24pr (anterior 24 prime), Caudate, p32pr (posterior 32 prime), 8C (Brodmann area 8 component C), PEF (prefrontal eye field), IFJp (inferior frontal junction area/posterior), 6r, PFcm (Brodmann area 40 PFcm complex), PHA2 (parahippocampal area 2), PHA3 (parahippocampal area 3), RI (retroinsular), STSdp (superior temporal sulcus dorsal posterior), and thalamus (Figure 3) (Baker, Burks, Briggs, Conner, et al.,^2018^; Baker et al., 2018a).

### 3.2 FST and PH are connected to extraoccipital areas

Based off our observation that FST and PH demonstrated the greatest coactivation with extraoccipital areas outside of the lateral occipital ROIs, we performed deterministic tractography to test the structural connectivity between FST and PH and their coactivated areas.

Area FST, located in the anterior portion of the LOL just posterior to MT gyrus, demonstrated a variety of structural connections to extravisual cerebrum mainly through long‐range superior longitudinal fasciculus (SLF) fibers and short local association fibers to the TPOJ1 (Figure 5). We found that in a small proportion of subjects, it also demonstrated connections to area 44 in the lateral frontal lobe, to area FOP4 along the inner surface of the pars opercularis of the inferior frontal gyrus, to area 6r in the premotor region, and to the TPOJ1 along the inferior portion of the parietal lobule.

**FIGURE 4:**
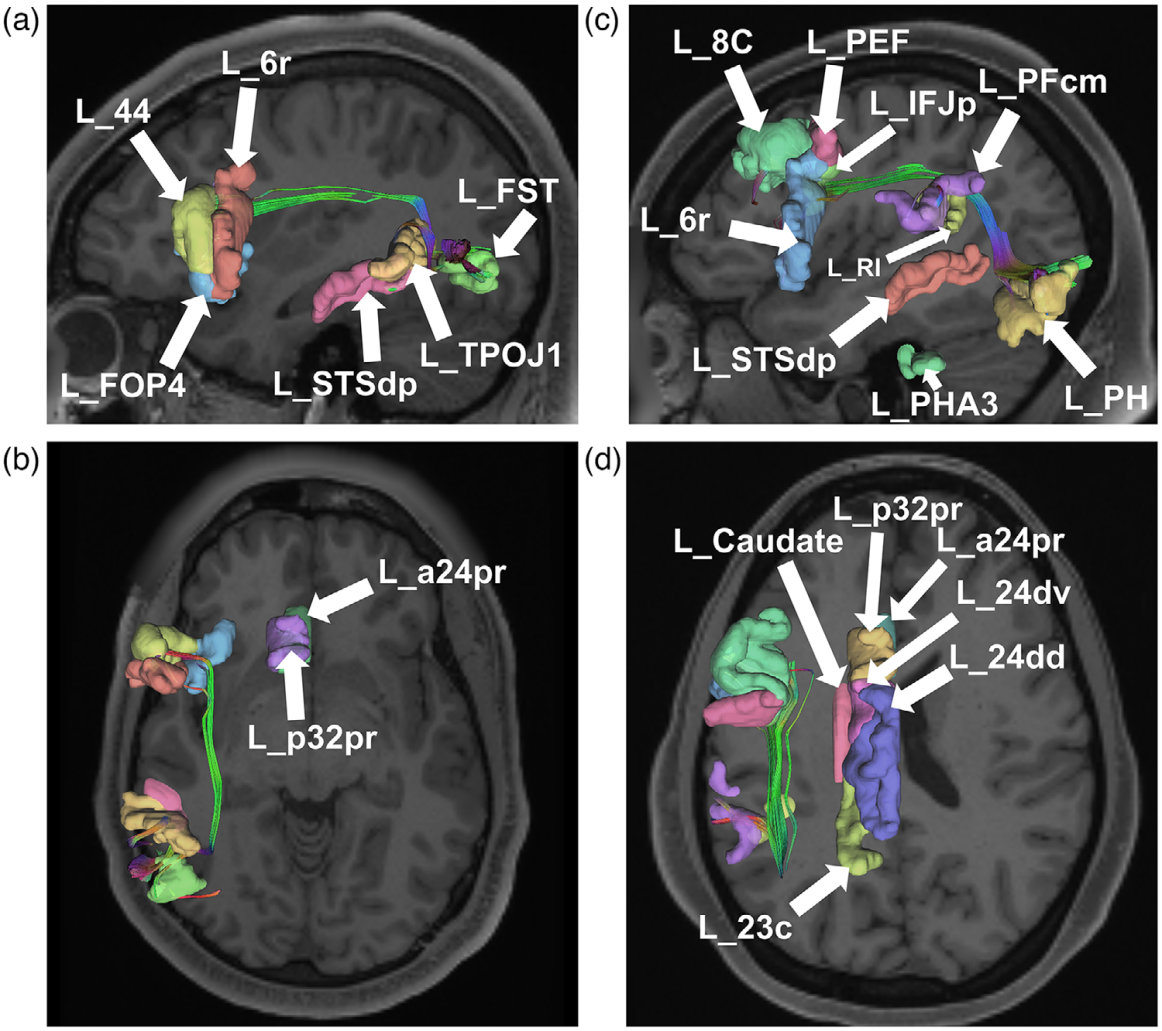
Tractographic model of areas fundus of the superior temporal sulcus (a, b) and parietal area H (c, d) and their functionally coactivated parcellations as shown on T1‐weighted magnetic resonance images in the left (L) cerebral hemisphere in sagittal and axial slices. Tractography presented is from a single healthy individual. Note that the figures are for visualization only and include parallax error. 8c, Brodmann area 8 component C; PEF, prefrontal eye field; IFJp, inferior frontal junction area/posterior; 6r, rostral premotor area 6; 44, Brodmann area 44; FOP4, frontal operculum 4; p32pr, posterior 32 prime; a32pr, anterior 32 prime; a24pr, anterior 24 prime; 24dv, ventral area 24d; 24dd, dorsal area 24d; 23c, Brodmann area 23 component C; 23d, Brodmann area 23 component D; PFcm, Brodmann area 40 PFcm complex; RI, retroinsular; STSdp, superior temporal sulcus dorsal posterior; PHA2, parahippocampal area 2; PHA3, parahippocampal area 3; TPOJ1, temporo–parieto–occipital junction 1; FST, fundus of the superior temporal sulcus; PH, parietal area H.

Area PH, located in the anteroinferior LOL just lateral to the occipitotemporal sulcus, also demonstrated long‐range SLF connections to extravisual cerebrum as well as short local association fibers to lateral parietal regions (Figure 4). Connections were identified from area PH to area 6r in the premotor region, areas 8C, and IFJp, and to lateral parietal regions PFcm, RI, and STSdp via U‐shaped local association fibers. There were no notable differences between left‐ and right‐sided connectivity of FST and PH (Table 3).

**TABLE 3:** Table showing the average number of tracts between identified parcels according to structural tractography analyses on healthy subjects from the Human Connectome Project. Parcellations are presented within Human Connectome Project nomenclature

Cortical regions	Number of subjects identified with connection (%)	Average connections weighted by all subjects (L+R/2)	Number of connections averaged by only identified subjects			
			Average connections (L+R/2)	L	R	LI
From FST						
6r	L = 1	0.16	3.67	3	4.33	–0.18
	R = 3					
44	L = 1	0.08	2.13	3	1.25	0.41
	R = 4					
a24pr	L = 0	0	0	0	0	0
	R = 0					
FOP4	L = 3	0.16	2.25	3	1.5	0.33
	R = 4					
p32pr	L = 0	0	0	0	0	0
	R = 0					
STSdp	L = 0	0.15	3.75	0	7.5	–1
	R = 2					
TPOJ1	L = 25	7.33	14.68	18.7	10.65	0.27
	R = 23					
From PH						
STSdp	L = 4	0.06	0.75	1.5	0	1
	R = 0					
RI	L = 2	0.07	1.75	3.5	0	1
	R = 0					
PHA3	L = 20	6.85	20.6	12.65	28.5	–0.39
	R = 15					
PHA2	L = 1	0.33	3.7	1	6.4	–0.73
	R = 5					
PFcm	L = 4	0.88	10.75	21.5	0	1
	R = 0					
PEF	L = 2	0.28	7	13	1	0.86
	R = 1					
p32pr	L = 1	0.11	5.5	11	0	1
	R = 0					
IFJp	L = 6	1.96	19.4	28.7	10	0.48
	R = 2					
a24pr	L = 0	0	0	0	0	0
	R = 0					
24dd	L = 1	0	0	0	0	0
	R = 0					
24dv	L = 0	0.01	0	0	0	0
	R = 0					
23d	L = 0	0	0	0	0	0
	R = 0					
23c	L = 0	0	0	0	0	0
	R = 0					
8C	L = 3	0.14	2.33	1.33	3.33	–0.43
	R = 3					
6r	L = 6	1.18	9.75	8.33	11.2	–0.15
	R = 6					
Thalamus	L = 4	0.90	13.63	7.25	20	–0.45
	R = 3					
Caudate	L = 4	0.12	2.25	2.5	2	0.11
	R = 1					

**FIGURE 5:**
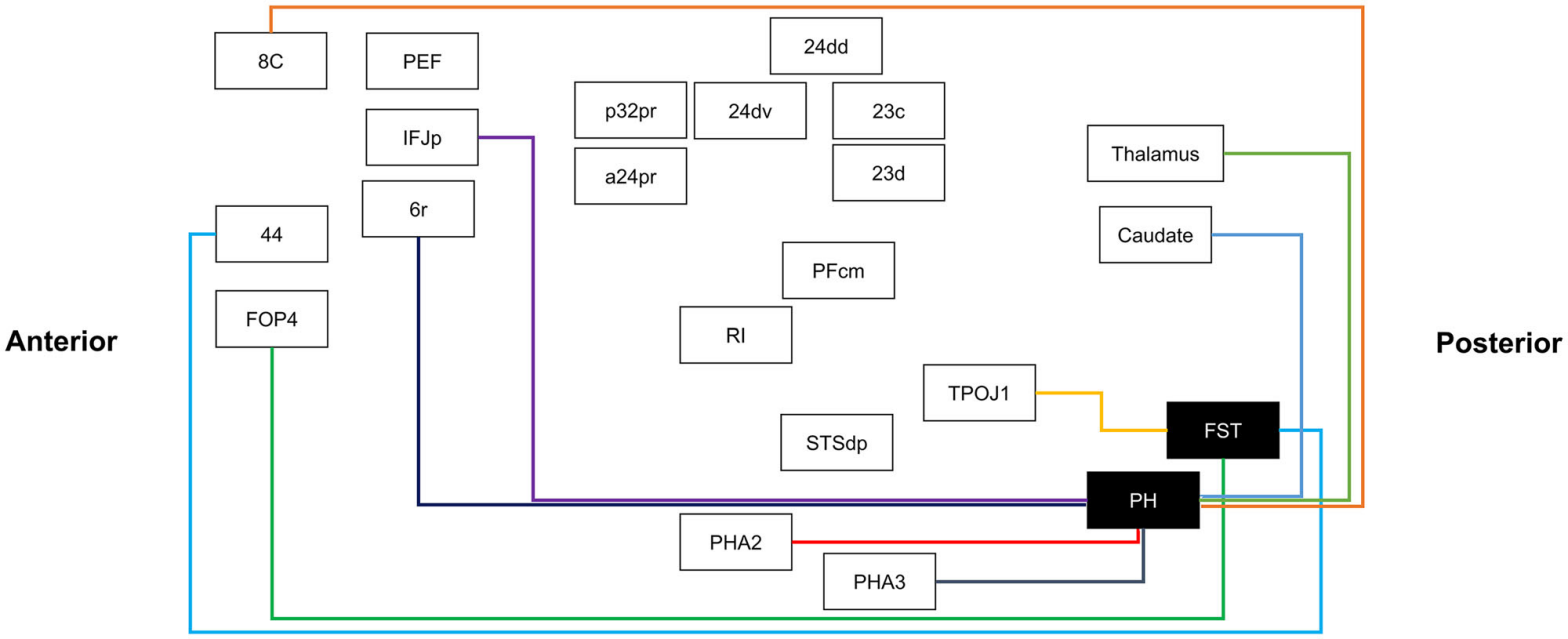
A simplified connectogram of the white matter connections identified between individual parcellations found during fiber tracking analysis across 50 healthy subjects. The diagram presents brain regions in the anterior–posterior direction when going clockwise starting at area fundus of the superior temporal sulcus. 8c, Brodmann area 8 component C; PEF, prefrontal eye field; IFJp, inferior frontal junction area/posterior; 6r, rostral premotor area 6; 44, Brodmann area 44; FOP4, frontal operculum 4; p32pr, posterior 32 prime; a32pr, anterior 32 prime; a24pr, anterior 24 prime; 24dv, ventral area 24d; 24dd, dorsal area 24d; 23c, Brodmann area 23 component C; 23d, Brodmann area 23 component D; PFcm, Brodmann area 40 PFcm complex; RI, retroinsular; STSdp, superior temporal sulcus dorsal posterior; PHA2, parahippocampal area 2; PHA3, parahippocampal area 3; TPOJ1, temporo–parieto–occipital junction 1; FST, fundus of the superior temporal sulcus; PH, parietal area H.

Our findings of the connectivity of areas FST and PH support our previous greater characterizations of the structural connectivity of the lateral occipital cortex (Baker et al., 2018b; Conner et al., 2018). Namely, areas FST and PH demonstrate wide connectivity outside of known visual cerebrum and demonstrate possible connections that can provide visual information into semantic and related networks (discussed further in Section 4). A schematic showing the average tract density between these parcellations is shown in Figure 5. A table showing the average number of tracts between these parcellations is shown in Table 3.

## 4 DISCUSSION

In this study, we discuss the results of a coordinate‐based ALE meta‐analysis of activated LOL parcellations in relation to its extraoccipital connections demonstrated outside of the visual network. We found that FST and PH exhibited the greatest coactivation in fMRI studies of healthy controls suggesting their possible hub‐like roles in transmitting visual information to the nonvisual cerebrum for higher order functions. Following the identification of FST and PH, we utilized DSI‐based fiber tractography to determine how their structural interconnectedness with other cortical regions may support their nonvisual functional roles, especially in language and reading. Interestingly, these parcellations are far more highly interconnected with other cortical regions compared to their neighboring areas in the LOL, which has a more modular organization (Baker et al., 2018b; Krubitzer & Kaas, 1990). For instance, areas LO1 and LO2 mostly contain structural connections to *local* parcellations through short association bundles (Baker et al., 2018b). Previous research using task‐based fMRI data and studies on primate brains suggested two mediotemporal ROIs, MT and MST, facilitate the biggest role in the integration of visual information (Ilg, 2008). However, emerging technologies, analytic assessments, and white matter fiber tracking have facilitated the study of larger data sets with finer precision to characterize the parcellations of the lateral occipital system in more detail (Baker et al., 2018b; Glasser et al., 2016). Using coactivation and structural connectivity studies, we investigated the roles that these regions play in visual processing and identified areas that communicate with regions outside of the occipital lobe and visual network. Our findings suggest that MT and MST individually demonstrate only modest communication with the rest of the cortex, indicating that areas FST and PH are highly connected to nonvisual cerebrum, which we believe warrants further investigation in humans.

Our finding that areas FST and PH demonstrated the greatest number of extraoccipital cortical connections and coactivation outside of the occipital lobe supports the “small‐world network” architecture of the lateral visual system (Dushanova & Tsokov, 2020; Shi et al., 2015). This posits that various nodes connect to hub‐like regions that are designed to integrate information and transmit the input to other hubs throughout the cortex for global processing and higher order tasks such as for reading, semantic processing, movement, and coordination.

### 4.1 Implications for visuospatial disorders

There is a topological overlap between FST and PH and areas in the dyslexia literature that demonstrate atypical functioning. Imaging studies on dyslexics have found abnormalities within the temporooccipital area, suggesting their importance in the complex cognitive process of reading (Bailey et al., 2018; Martin et al., 2016; Schurz et al., 2015; Ting et al., 2017). In fact, several studies have demonstrated that reading difficulties in dyslexia may be due to visual processing deficits, rather than phonological problems (Werth, 2021b). Even pediatric patients with severe dyslexia demonstrate the ability to read pseudowords if given sufficient fixation time, suggesting they may require greater time for temporal summation, a process that occurs early in visual processing (Cheng et al., 2018; Werth, 2019, 2021a, 2021b). Furthermore, it has been demonstrated that the fusiform gyrus plays a significant role in the ability to recognize words and pseudowords (Werth, 2021b). Several studies have also identified structural and functional impairments within the temporooccipital regions in individuals with dyslexia including reduced gray matter volume (Kronbichler et al., 2008), hypoactivation during reading tasks (Martin et al., 2016; Richlan et al., 2011, 2010), and elevated concentration of harmful metabolites related to diminished white matter integrity (Kossowski et al., 2019). Consequently, given the highly modular organization of the LOL, we believe that areas FST and PH should be studied as potential pathoanatomical hubs underlying the atypical integration of visual and nonvisual information seen in dyslexia.

The convergence in the literature linking abnormalities in dyslexia in the temporooccipital region, which co‐opt FST and PH, may also underlie why there are cross‐network abnormalities in dyslexia (Bailey et al., 2018). If FST and PH serve as hub areas integrating information from multiple different functional systems, then their absence could help explain why dyslexic patients have trouble integrating multiple numerous aspects of reading—that is, visual processing and phonological, semantic, and syntactic information (Rüsseler et al., 2007). Nonetheless, there are also many other cortical regions serving as key hubs required for the processing of complex functions like language and reading. Examples of this include the posterior cingulate cortex and the inferior frontal gyrus that have roles in higher cognition and language manipulation, and also have similar characterizations in dyslexia (Bailey et al., 2018; Finn et al., 2014; Hagoort, 2005).

### 4.2 Connectivity of area FST to nonvisual areas

Based on the ALE and tractography results, FST demonstrates functional and structural connections between area FST and the lateral frontal lobe (areas 44, 6r, and FOP4) and TPOJ1. FST also shows coactivation with two of the parcellations in the anterior cingulate gyrus, a24pr and p32pr, though structural connections were not consistently identified to these regions during fiber tracking analysis.

The parcellations connected to FST in the anterior cingulate gyrus (a24pr and p32pr) and area FOP4 have been shown in previous studies to play a role in gestalt language retrieval (Palomero‐Gallagher et al., 2009; Shackman et al., 2011). Additionally, area p32pr and the TPOJ1 have been shown to be involved in attention and decision‐making during language‐based tasks (Mars et al., 2012; Shackman et al., 2011; Vrticka et al., 2013). This extensive connectivity to language centers responsible for attention and retrieval suggests that area FST plays a critical role in supplying visual input for language comprehension—the semantic retrieval of language and reading (Fiez et al., 1999).

Upon considering the emphasis of FST on providing visual input for language functions and reading, we must also address its connections with area 44 and the rostral portion of area 6: two areas often associated with Broca's speech production area (Petrides & Pandya, 1999). While primarily studied as the region responsible for facial and laryngeal muscle control, the literature also suggests these areas play a role in the cognitive processes required for the production of fluent speech, with related damage leading to speech apraxia (Ding et al., 2014; Petrides & Pandya, 1999; van Schie et al., 2006). Though it may be difficult to interpret the role FST plays in speech production, connections to areas 44 and 6r could indicate that FST plays a role in speech cognition and fluency.

### 4.3 Connectivity of area PH to nonvisual areas

The functional and structural connections of area FST suggest that it plays a role in language processing and retrieval. Meanwhile, area PH demonstrates structural connectivity to supplementary motor areas (8C and IFJp), lateral parietal regions (PF, PFcm, RI, and STSdp), and part of the lateral frontal lobe (6r). Area PH also shows coactivation with the motor division of the anterior cingulate gyrus (24dv and 24dd), part of the cognitive division of the anterior cingulate gyrus (p32pr), and a supplementary motor area (PEF), though structural connections were only identified with PEF.

Previous studies conducted on the lateral parietal regions, PF and RI, and STSdp, as well as lateral frontal areas 8C and PEF, and the supplementary and cingulate eye field (SCEF), suggest these parcellations play an important role in the integration of motion, audiospatial, and visuospatial information to direct visual attention and oculomotor planning and saccadic control required in reading (Amiez & Petrides, 2009; Deen et al., 2015; Mars et al., 2012; Petrides & Pandya, 1999; Watson et al., 2014).

Additionally, the anterior cingulate areas 24dd and 24dv act as motor planning centers for proximal muscles of the lower and upper limbs, respectively, for coordinated, complex movements (Palomero‐Gallagher et al., 2009; Shackman et al., 2011). Functional coactivation with motor and spatially oriented cortical regions, therefore, suggests that area PH may play a role in supplying visual input to motor planning centers to aid in coordination and the execution of complex movements through grasping and smooth eye pursuit. Interestingly, abnormal oculomotor control and eye tracing can be used as a screening method for dyslexia. This may be the result of abnormal connectivity between FST and eye movement regions (Nilsson Benfatto et al., 2016). However, consistent structural connections were not identified between these regions and therefore further study is necessary to understand how these functions are facilitated according to underlying structural–functional connectivity with likely greater analytical specificity.

Similar to area FST, PH demonstrates functional and structural connectivity to the lateral frontal lobe (6r and IFJp) and the cognitive portion of the anterior cingulate gyrus (p32pr), which have been linked to language processing and the integration of top‐down and bottom‐up processing of language (Amiez & Petrides, 2009; Calvert & Campbell, 2003; Petrides & Pandya, 1999; Shackman et al., 2011). These connections, in conjunction to cortical regions involved in spatial integration and oculomotor control, suggest that area PH plays an additional role in visual language gestalt, semantics, and the visual feedback to oculomotor centers required for reading (Paulesu et al., 2003; Rossell et al., 2001; Siok et al., 2004).

### 4.4 Significance of findings

We provide evidence of critical hub regions within the LOL, connecting the visual system to nonvisual parts of the cerebrum. Previous studies investigating the integration of visual information indicated that MT and MST played the most significant role; however, these studies largely relied on macaques for data collection (Krubitzer & Kaas, 1990; Lui & Rosa, 2015). By analyzing fMRI studies of human brains with ALE meta‐analytic methodology, our results indicate that MT and MST have only minimal connections to extraoccipital areas. Instead, areas FST and PH show the greatest connections to extraoccipital areas suggesting their role as possible hub areas for information processing from multiple functional systems.

It appears that FST and PH act as hubs in the lateral occipital network, collecting and relaying information from within the lateral visual system to extravisual parcellations responsible for various multinetwork functions, such as spatial, motor, and language functions. This supports the organization of the lateral occipital system as a small‐world network. Specifically, parcellations within the LOL display extensive internal communication, while areas FST and PH act as a bridge between the lateral visual and extravisual networks.

These findings are indirectly supported by imaging studies that demonstrate abnormalities in multiple functional networks in individuals with dyslexia, including visual, attention, and cingulo‐opercular networks (Bailey et al., 2018). This may be attributable to the extensive functional and structural connections areas PH and FST form outside the visual system (Baker et al., 2018b). Furthermore, similar reading and language deficits have been commonly linked to areas we identified as having strong visual to nonvisual area connectivity (Ilg, 2008). Future research can attempt to better quantify this relationship using graph theory with whole‐brain connectivity analyses in both adults and children (Finn et al., 2014). Research efforts can also be directed toward identifying associated functions of FST and PH, and their connections throughout the cortex.

### 4.5 Limitations

Although our study provided an extensive topographical model of the lateral visual system, it is not without its limitations. Coordinate‐based meta‐analyses allow the procuring of foci reported from numerous different experiments that can improve study power and the ability to generate hypotheses for further discussion. However, this type of analysis is also inherently limited by the quality of the data reported in available studies due to common heterogeneity in study designs and biases in selected studies. While numerous revisions of this method have been previously described, the ALE is subject to possible publication bias like most meta‐analyses and may require additional analyses to control for unreported information (Acar et al., 2018; Eickhoff et al., 2012).

Furthermore, we elucidated the hubs of the visual network that we identified in HCP nomenclature, which delineates 180 distinct cortical areas across two hemispheres allowing us to use complex computational algorithms to describe the neuroanatomical basis of the LOL with great precision (Glasser et al., 2016). However, this finer subdivision of cortical areas may contribute to a brain atlas concordance problem, which has traditionally been attributed to a neuroanatomical nomenclature problem where heterogenous classifications may be given to structurally and functionally homologous brain areas (Bohland et al., 2009). Fortunately, a coordinate‐based ALE meta‐analytic approach allowed us to overcome some of these difficulties in order to compare our findings to the literature and interpret classical neuroanatomical maps with a degree of freedom permitted by the more general topographic classifications. Future studies should embrace more specific topographic nomenclature to report findings and also consider reporting in a common, established vernacular, such as the HCP nomenclature, to systematically improve our neuroanatomical understanding over future refinements.

We discussed the role of FST and PH in the context of langue and reading disorders while also excluding studies related to the behavioral paradigms including visual processing. These excluded studies may have further explained the role of the LOL in language‐related visuospatial processing and thus represent a limitation of our methods. However, it is important to note that the BrainMap database includes an intricate search software that allowed us to first screen all studies related to visual processing to identify cortical regions in the visual network and only then subsequently filter out studies solely focused on visual processing to better understand the nonvisual functions of possible hub areas identified (Laird et al., 2009). This exhaustive search strategy in addition to analyzing coactivation patterns of selected coordinates with strict cluster interference algorithms was to ensure appropriate screening of all studies according to our study objectives before subsequent filtering and also to ensure statistically likely convergence patterns were not likely due to chance alone. Similarly, our analyses were focused on healthy HCP subjects who commonly demonstrate leftward lateralization of language networks; however, dyslexic patients may demonstrate a more rightward asymmetry (Finn et al., 2014; Milton et al., 2021). Hub areas undoubtedly integrate cross‐network information for complex functions; however, the explicit implications of FST and PH in reading and language processing among other nonvisual complex functions in humans require further controlled, prospective investigations mapping whole‐brain networks.

It is important to note that combined studies such as ours that utilize both structural tractography and functional coactivation maps together are able to better investigate connectomic information compared to either of these modalities alone, but are inherently limited by the quantitative limitations of tractographic analyses (Campbell & Pike, 2014). For instance, in Table 3, a great deal of variability can be seen in the relative fiber volume within identified subjects. While this may raise concerns about the relative strength and therefore importance of specific connections, it must be reminded that the relative strength of tractographic analyses is the ability to qualitatively demonstrate the nature of the more common white matter connections between ROIs, which can clarify specific connections that may support possible known functional relevance in connected regions or provide areas of basis for future study (Briggs et al., 2022; Essayed et al., 2017). Therefore, our quantitative analyses should be interpreted cautiously within the context of these known limitations, and further and more rigorous analyses on these connections are necessary to better clarify their relative strength and interindividual differences.

In conclusion, we identified two hub‐like regions of the LOL that form extensive connections with nonvisual cerebral areas. Elsewhere, abnormal activation of FST and PH has been described to illicit multinetwork disturbances that affect both visual and nonvisual functions, such as language and reading. FST and PH should be included in future studies as possible covariates to include in the modeling of the atypical mechanisms of language‐ and reading‐related multinetwork disturbances, such as in dyslexia. Further studies should refine our model with whole‐brain analyses in healthy individuals as well as in individuals with visuospatial disorders to better understand the implications of FST and PH as key hub areas for nonvisual cerebrum. Importantly, the current study demonstrates that ALE meta‐analytic software targeting functional data in large data sets can be applied to other cortical regions as well throughout the brain to determine possible hub areas, and subsequent structural tractography can be applied to understand how their structural interconnectedness may support their functional roles in integrating complex information across multiple brain networks.

## CONFLICT OF INTEREST STATEMENT

M. E. Sughrue is the Chief Medical Officer of Omniscient Neurotechnology, and no products related to this were discussed in this paper. The other authors declare no conflicts of interest.

### PEER REVIEW

The peer review history for this article is available at https://publons.com/publon/10.1002/brb3.2945.

## Data Availability

All data discussed in the current work have been provided. Additional data not provided can be made available by the authors upon request.
